# The positive effects of running exercise on hippocampal astrocytes in a rat model of depression

**DOI:** 10.1038/s41398-021-01216-x

**Published:** 2021-02-01

**Authors:** Yue Li, Yanmin Luo, Jing Tang, Xin Liang, Jin Wang, Qian Xiao, Peilin Zhu, Kai Xiao, Lin Jiang, Xiaoyun Dou, Chunxia Huang, Yuhan Xie, Yong Tang

**Affiliations:** 1 Department of Histology and Embryology, Chongqing Medical University, 400016 Chongqing, P. R. China; 2 Laboratory of Stem Cells and Tissue Engineering, Chongqing Medical University, 400016 Chongqing, P. R. China; 3 Department of Physiology, Chongqing Medical University, 400016 Chongqing, P. R. China; 4 Department of Pathophysiology, Chongqing Medical University, 400016 Chongqing, P. R. China; 5 Department of Radioactive Medicine, Chongqing Medical University, 400016 Chongqing, P. R. China; 6 Lab Teaching & Management Center, Chongqing Medical University, 400016 Chongqing, P. R. China; 7 Institute of Life Science, Chongqing Medical University, 400016 Chongqing, P. R. China

**Keywords:** Molecular neuroscience, Depression

## Abstract

Running exercise has been shown to alleviate depressive symptoms, but the mechanism of its antidepressant effect is still unclear. Astrocytes are the predominant cell type in the brain and perform key functions vital to central nervous system (CNS) physiology. Mounting evidence suggests that changes in astrocyte number in the hippocampus are closely associated with depression. However, the effects of running exercise on astrocytes in the hippocampus of depression have not been investigated. Here, adult male rats were subjected to chronic unpredictable stress (CUS) for 5 weeks followed by treadmill running for 6 weeks. The sucrose preference test (SPT) was used to assess anhedonia of rats. Then, immunohistochemistry and modern stereological methods were used to precisely quantify the total number of glial fibrillary acidic protein (GFAP)^+^ astrocytes in each hippocampal subregion, and immunofluorescence was used to quantify the density of bromodeoxyuridine (BrdU)^+^ and GFAP^+^ cells in each hippocampal subregion. We found that running exercise alleviated CUS-induced deficit in sucrose preference and hippocampal volume decline, and that CUS intervention significantly reduced the number of GFAP^+^ cells and the density of BrdU^+^/GFAP^+^ cells in the hippocampal CA1 region and dentate gyrus (DG), while 6 weeks of running exercise reversed these decreases. These results further confirmed that running exercise alleviates depressive symptoms and protects hippocampal astrocytes in depressed rats. These findings suggested that the positive effects of running exercise on astrocytes and the generation of new astrocytes in the hippocampus might be important structural bases for the antidepressant effects of running exercise.

## Introduction

Depression is a common mental disorder with an increasing mortality rate^1^ and understanding the mechanisms of depression and antidepressant therapeutics is crucial. Numerous studies have demonstrated that the etiology of depression includes not only stress-induced mental issues but also stress-induced pathological lesions, particularly in encephalic regions^2^, especially the hippocampus and prefrontal cortex (PFC). The hippocampus is associated with cognition and memory^3^, and it has been reported to modulate depressed mood^4,5^. Postmortem studies of depression patients have demonstrated that hippocampal volume is decreased in these patients compared to control subjects^6,7^. Similar findings have been reported in animal models of depression^8,9^. Furthermore, Czéh et al. have found that the stress-induced reduction in astroglial number is highly correlated with a reduction in hippocampal volume^10^. Astrocytes are the most abundant glial cells in the central nervous system (CNS) and account for 20–40% of all glial cells in the human brain^11–13^. Increasing evidence has indicated that astrocyte pathology in several encephalic regions might contribute to the mechanism of depression in both depressed subjects and animal models^14–18^. Human postmortem studies have revealed that the density of astrocytes in the hippocampus is lower in depressed subjects than control subjects^19,20^. Furthermore, using stereological methods, Czéh et al. observed astroglial loss in the hippocampus of male tree shrews after long-term psychosocial stress^10^. The hippocampal formation is a well-organized structure that has a complex anatomical organization and cellular composition, and its subregions have specific structural connectivity and functional roles^21^. Mild chronic psychogenic stress typically leads to dendrite retraction in CA3 pyramidal neurons, but not CA1 pyramidal neurons or granule cells in the dentate gyrus (DG)^22,23^. The CA1 becomes susceptible to acute stress after stress sensitization, and chronic stress can reduce synaptic plasticity in both the CA1 region and DG^24^. Adult neurogenesis, which has been suggested to play an important role in depression, specifically occurs in the DG^25^. All of these findings indicate that different subfields of the hippocampus might respond differently to stress. However, the change in the number of astrocytes that occurs in each hippocampal subregion in depressed animals is unknown. Moreover, new glial cells are produced in the CNS of adult mammals through gliogenesis^26^, and the integration of newborn cells into the neuronal network is important for the function of the CNS^27^. However, whether changes in newborn astrocytes in the hippocampal subregions affect astroglial loss in depressed animals is still unclear. To explore these issues, an unbiased stereological technique was used to study the total number of astrocytes, and immunofluorescence was used to study the density of bromodeoxyuridine (BrdU)^+^/glial fibrillary acid protein (GFAP)^+^ cells in each of three hippocampal subregions (the CA1 region, CA2/3 region, and DG) in a chronic unpredictable stress (CUS) rat model of depression.

Running exercise, as a simple behavioral therapy, has been widely demonstrated to have antidepressant effects^28^. Studies have reported that physical exercise is associated with a greater reduction in depressive symptomology than no treatment or placebo in depression^29^. Our previous studies have demonstrated that treadmill exercise can improve the depressive-like behaviors induced by CUS in rats^30,31^. However, the mechanisms underlying these antidepressant effects of running exercise are still unknown. Several studies have found that the improvements in depressive symptoms^20^ and depressive-like behaviors^10,32,33^ induced by antidepressant therapy are accompanied by changes in astrocytes. Furthermore, Cui et al. showed that astrocyte-specific loss of Kir4.1 in the lateral habenula can alleviate the depression-like symptoms of depressed rats^16^, furthermore, Cao et al. showed that selective activation of astroglial Ca^2+^ signaling can stimulate endogenous ATP release from astrocytes and induce antidepressant-like effects in depressed mice^17^. These studies indicate that astrocytes might play an extremely important role in the mechanism of antidepressant therapy. Studies have reported that running exercise can increase the density of astrocytes in the CA1 region of the hippocampus^34^, parietal cortex and dorsolateral striatum^35^ in normal rats and in the hippocampus of rats with type 1 diabetes mellitus (T1DM)^36^. Moreover, Ehninger and Kempermann^37^ found that running exercise can increase the density of BrdU^+^/S100β^+^ cells in the cingulate, motor and visual cortex in normal mice. Similarly, running exercise has been shown to increase the number of newborn astrocytes in the hippocampus in normal adult rats^38^. These studies indicated that running exercise can increase the density of astrocytes and promote the generation of new astrocytes in normal rodents and rats with T1DM, but whether running exercise can reverse astroglial loss in the hippocampus in depression is poorly understood.

In the current study, we hypothesized that running exercise alleviates depression through increasing the number of astrocytes and enhancing the generation of new astrocytes in the hippocampus of rats with CUS-induced depression. To verify this hypothesis, the antidepressant effects of running exercise were first assessed with the sucrose preference test (SPT). Then, stereological and immunofluorescence techniques were used to investigate astrocyte and newly generated astrocytes in three subregions of the hippocampus.

## Materials and methods

### Animals

Sixty male Sprague-Dawley rats (weight 150 ± 10 g) from Chongqing Medical University (Chongqing, PR China) were housed in groups (5 rats per cage) at a constant temperature (22 ± 2 °C) on a 12-h light/dark cycle (7 AM–7 PM) and provided free access to food and water. During the animal experiment, the animals in each group were treated by the investigators without blinding. All procedures were conducted in accordance with the National Institutes of Health Guide for the Care and Use of Laboratory Animals and were consistent with the Chongqing Medical University Care and Use of Laboratory Animals guidelines.

### CUS intervention

**Fig. 1:**
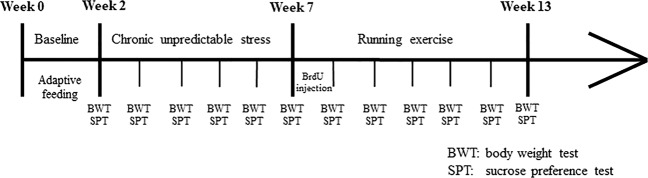
Behavioral experiment timeline. The experiment lasted a total of 13 weeks. The animals were allowed to habituate to the housing conditions for 2 weeks before any experiments were initiated. The CUS rats were subjected to a sequence of 10 different stressors (two stressors per day for 5 weeks). The animal in the CUS/running group ran on a six-lane motorized treadmill for 6 weeks after CUS. Body weight and sucrose preference were assessed each weekend. CUS chronic unpredictable stress, BWT body weight test, SPT sucrose preference test.

All rats were allowed to habituate to the housing conditions for 2 weeks. Next, 60 rats were randomly divided into the control group (*n* = 23) (nonstressed rats) and the CUS group (*n* = 37) (stressed rats). Each rat in the CUS group was subjected to a sequence of 10 different stressors (cold/heat stress, light changes (on/off), noise, food or/and water deprivation, empty bottle, wet bedding, electric shock, 1-h restraint, tail pinching and cage tilting) for 5 weeks (two stressors per day) (Fig. 1, Table 1). This procedure was adapted from Willner et al.^39^, Banasr et al.^40^, and Seney et al.^41^. The control group was housed under normal conditions. The effects of stress on the hedonic state of the rats were assessed with the SPT and body weights were measured each weekend^39^. Then, the rats in the CUS group were randomly divided into the CUS/standard group (*n* = 17) (stressed rats not subjected to running exercise) and the CUS/running group (*n* = 20) (stressed rats subjected to running exercise).

**Table 1:** The schedule of CUS model intervention.

Time	Monday	Tuesday	Wednesday	Thursday	Friday	Saturday	Sunday
Week 1	Wet bedding cage tilting	Water deprivation light off	Electric shock empty bottle	Restraint light on	Tail pinching noise	Food deprivation heat stress	BWT SPT
Week 2	Wet bedding noise	Cold stress empty bottle	Cage tilting tail pinching	Water deprivation food deprivation	Electric shock heat stress	Light off restraint	BWT SPT
Week 3	Light on tail pinching	Cage tilting light off	Water deprivation restraint	Wet bedding empty bottle	Noise electric shock	Heat stress food deprivation	BWT SPT
Week 4	Wet bedding water deprivation	Electric shock empty bottle	Light off cold stress	Cage tilting restraint	Noise tail pinching	Food deprivation light on	BWT SPT
Week 5	Noise food deprivation	Water deprivation heat stress	Electric shock light on	Wet bedding tail pinching	Light off empty bottle	Cage tilting restraint	BWT SPT

*CUS* chronic unpredictable stress, *BWT* body weight test, *SPT* sucrose preference test.

### Running exercise protocol and BrdU injection

In the CUS/running group, the animals ran on a six-lane motorized treadmill for 20 min per day, 5 days per week for 6 weeks. The running speed was gradually increased from 10 to 20 m/min during the first week and then maintained at 20 m/min for the next 5 weeks^42–44^. The control group and the CUS/standard group were housed under conditions are not subjected to running exercise. BrdU (20 mg/mL, dissolved in 0.9% saline solution; B5002, Sigma, USA) was injected (5 ml/kg, i.p.) once a day for 7 consecutive days, during the first week of running exercise^45–47^.

### Body weight measurement

The body weights of the rats in each group were obtained before sucrose consumption every weekend.

### SPT

The SPT was used in this study to assess anhedonia. The SPT was performed each weekend at 8:30 AM after body weight measurement. During the test, the rats were allowed access to two bottles of water, the one containing fresh water and the other containing 1% sucrose solution, for 24 h and left undisturbed. The bottle containing the sucrose solution was placed randomly on the left or right side of the cage. This protocol was adapted from Willner et al.^39^ and Banasr et al.^40^. The sucrose preference rate was calculated as sucrose consumption/total liquid consumption × 100%.

### Perfusion and tissue preparation

During the following processes, all the experiments and data analyses were performed blind to treatment conditions. Five random rats from each group were deeply anesthetized by i.p. injection of 1% pentobarbital sodium (4 ml/kg) and perfused with 4% paraformaldehyde. The brains were removed and divided into two hemispheres along the sagittal suture. The right or left hemisphere of each rat brain was selected at random. After perfusion, the hemispheres were fixed in 4% paraformaldehyde for at least 24 h and then immersed in increasing concentrations (10, 20, and 30%) of sucrose in 0.1 M PBS (pH 7.4) until equilibrium was reached. Then, hemispheres were rapidly frozen at −60 °C and cut into 50-µm thick serial sections on a cryostat microtome (CM1860, Leica, Germany). The sections were stored at −20 °C after being rinsed with 0.01 M PBS and 75% alcohol. Every 6th section containing the hippocampus was selected for analysis in this study. On average, 20 sections of the hippocampus were sampled per hemisphere.

### Cresyl violet staining and volume estimation

**Fig. 2:**
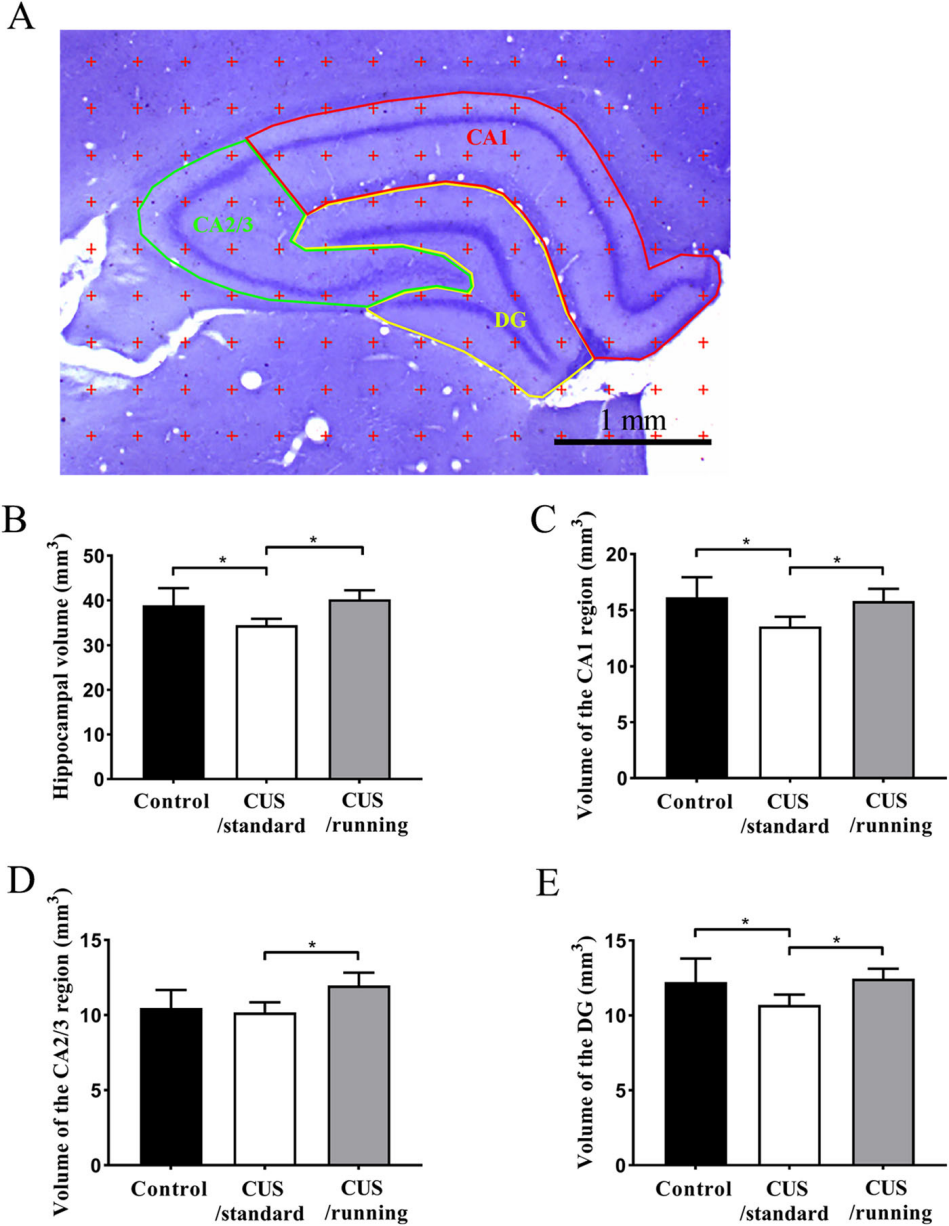
Illustrations of the method used to quantify the hippocampal volume and the positive effects of running exercise on the hippocampal volume of CUS rats. **A** An illustration of the stereological method used to estimate the hippocampal volume. The points located on the whole hippocampus and its subregions were counted. Bar = 1 mm. **B–E** The volume of the total hippocampus, the CA1 region, the CA2/3 region and DG in the control group (*n* = 5), the CUS/standard group (*n* = 5), and the CUS/cunning group (*n* = 5) (mean ± SD). Asterisk indicates *p* < 0.05. CUS chronic unpredictable stress.

The sections were stained with cresyl violet to help delineate the boundary of the hippocampus. Using an anatomical microscope (1.25×), the grid points located on the hippocampus were counted (Fig. 2A). The total hippocampus volume and the volume of each hippocampal subregion were estimated according to the Cavalieri’s principle^48,49^: V = t × a(p) × ΣP, where V is volume of the hippocampus or one of the hippocampal subregions, t is the tissue block thickness (0.6 mm), a(p) is the area associated with each grid point (0.09 mm^2^), and ΣP is the total number of grid points located on the hippocampus or one of the hippocampal subregions per rat.

### Immunohistochemistry

The sections were rinsed with 0.01 M PBS (pH 7.4) three times for 10 min each and thoroughly rinsed with 0.01 M PBS containing 0.3% Triton X-100 and 0.1% Tween-20 (PBS + T). Unless otherwise stated, all washes and incubations were performed in 6-well Netwell plates at room temperature (RT, 21–23 °C) with gentle agitation. Then, the sections were incubated in blocking buffer, 1% fetal bovine serum and 5% SP 9001-A (normal goat serum) diluted in PBS + T for 2 h at 37 °C followed by a rabbit anti-GFAP primary antibody (ab7260, Abcam, UK, 1:2000) diluted in the above-described blocking buffer for 72 h at 4 °C. After being washed with PBS + T, the sections were incubated with SP 9001-B (biotinylated goat anti-rabbit immunoglobulin, 1:20) diluted in PBS + T for 24 h at 4 °C and SP 9001-C (biotin-HRP-streptavidin) for 4 h at 37 °C. Then, the sections were transferred to diaminobenzidine (DAB) solution (ZLI-9018, ZSGB, China) for ~10 min. After being washed with distilled water and 0.01 M PBS, the sections were mounted onto glass slides and dehydrated by sequential immersion in a gradient of ethanol solutions (70, 80, 90, 100, and 100%, 5 min each) and xylene (three times, 10 min each). In order to make sure that the antibody penetrated the full height of the tissue, each stained section was carefully checked from the top to the bottom of the section.

### Stereological analysis

Based on the morphological features of the neurons in the hippocampus^50^, the CA1 region, CA2/3 region, and DG were delineated at ×2.5 magnification (Supplementary, Fig. 1A) with a stereological system (ZEISS, Germany). The area sampling fraction was set to 2%. Optical disector counting frames were placed in the delineated regions of the sections in a systematic, random fashion so that each section had an equal probability of being sampled and the interval between each section and each counting site was constant. The “guard zone”, which was set at 3 μm, was placed at the top surface of the sections, and the number of GFAP^+^ cells was counted through a depth of 15 μm below the “guard zone” (the height of the disector). The total number of astrocytes (N) was estimated according to the following equation (for the stereological sampling scheme, see Supplementary, Fig. 1B and Table 2): *n* = ΣQ^−^ × 1/*ssf* × 1/*asf* × 1/*hsf*, where ΣQ^−^ is the number of GFAP^+^ cells actually counted in the specimens, *ssf* is the section sampling fraction, *asf* is the area sampling fraction, and *hsf* is the height sampling fraction.

**Table 2:** Sampling scheme for the estimation of GFAP^+^ cell numbers.

	Control group	CUS/standard group	CUS/running group
Number of sections sampled			
CA1	16–19	16–19	17–19
CA2/3	26–30	27–32	26–33
DG	18–22	19–21	19–22
Section thickness (μm)			
CA1	23.18 ± 1.14	23.86 ± 1.12	23.56 ± 1.57
CA2/3	24.41 ± 1.55	24.48 ± 1.07	24.31 ± 1.09
DG	22.6 ± 1.16	22.65 ± 1.22	24.04 ± 2.51
Number of counting frames sampled			
CA1	234 (213–249)	222 (199–270)	257 (225–276)
CA2/3	161 (152–173)	144 (125–173)	180 (158–207)
DG	220 (199–234)	192 (170–216)	238 (223–265)
Number of GFAP^+^ cells sampled			
CA1	471 (435–496)	399 (311–490)	511 (430–581)
CA2/3	283 (249–330)	241 (213–290)	321 (280–393)
DG	496 (480–530)	385 (344–428)	537 (465–607)

Section thickness is represented as mean ± standard deviation (SD), whereas the number of counting frames and the numbers of sampled GFAP^+^ cells are represented as mean with range in parentheses.

*CUS* chronic unpredictable stress, *GFAP* glial fibrillary acidic protein.

### Immunofluorescence

After being washed with 0.01 M PBS and PBS + T, the free-floating sections were incubated in blocking buffer containing 1% fetal bovine serum, 5% SP 9001-A, and PBS + T for 2 h at 37 °C, and then stained with a rabbit anti-GFAP primary antibody for 48 h at 4 °C. The next morning, the sections were stained with a rat anti-BrdU primary antibody (ab6326, Abcam, UK, 1:500) for 48 h at 4 °C (after the treatment with 2 mol/l HCl for 50 min at 37 °C and boric acid for 20 min at room temperature). After the sections were washed with PBS + T for 30 min, the primary antibodies were detected with fluorophore-conjugated IgG. The sections were incubated at 37 °C for 2 h with goat anti-rabbit IgG DyLight 549 (Abbkine, USA) and goat anti-rat IgG DyLight 488 (Abbkine, USA) in the dark and then washed in the dark with PBS for 30 min. Then, the sections were mounted on gelatin-coated slides with anti-fade solution to reduce fluorescence quenching. Complete images of the hippocampus were obtained using laser scanning confocal microscopy (Nikon, Japan), and cells were counted after random and equidistant sampling in each subregions (NIS-Elements, 4.5). The complete image of immunofluorescence section was compared to the map section stained with DAPI to orientate the subregion of hippocampus. The numbers of BrdU^+^ cells, GFAP^+^ cells, and BrdU^+^/GFAP^+^ cells were estimated (for the sampling scheme, see Table 3).

**Table 3:** Sampling scheme for the estimation of BrdU^+^, GFAP^+^, and BrdU^+^/GFAP^+^ cell.

	Control group	CUS/standard group	CUS/running group
Number of sections sampled			
CA1	6–9	7–9	7–9
CA2/3	5–8	6–9	7–9
DG	7–9	7–9	8–10
Number of counting frames sampled			
CA1	46 (38–57)	45 (38–52)	45 (39–53)
CA2/3	39 (27–52)	40 (34–50)	42 (35–48)
DG	46 (36–52)	48 (41–54)	51 (44–64)
Number of BrdU^+^ cells sampled			
CA1	116 (87–138)	79 (50–115)	103 (78–122)
CA2/3	80 (55–89)	60 (46–95)	88 (78–105)
DG	111 (73–143)	88 (64–124)	142 (95–177)
Number of GFAP^+^ cells sampled			
CA1	892 (623–1243)	619 (418–763)	882 (620–1003)
CA2/3	770 (547–1061)	584 (415–746)	873 (565–1171)
DG	926 (640–1330)	647 (447–819)	1031 (735–1264)
Number of BrdU^+^/GFAP^+^ cells sampled			
CA1	39 (29–53)	16 (10–25)	33 (28–37)
CA2/3	24 (12–30)	17 (13–25)	33 (25–43)
DG	40 (26–64)	24 (11–36)	57 (51–63)

The number of counting frames and the numbers of sampled BrdU^+^ cells, GFAP^+^ cells, and BrdU^+^/GFAP^+^ cells are represented as mean with range in parentheses.

*CUS* chronic unpredictable stress, *GFAP* glial fibrillary acidic protein. *BrdU* bromodeoxyuridine.

### Statistical analyses

All data are expressed as the mean ± standard deviation (SD). All statistical analyses were conducted using SPSS 19.0 statistical software. The Shapiro–Wilk test was used to evaluate whether the data were normally distributed. Levene’s test was used to evaluate whether the variances were similar among the groups. The effects of CUS on sucrose preference and body weight were analyzed using repeated measures analysis of variance (ANOVA). All the data were normally distributed. If there was similar variance among groups, one-way ANOVA followed by LSD post-hoc test was used for analysis; otherwise, the Brown–Forsythe test and Tamhane T2 test were adopted for analysis. The coefficient of variation (CV) and coefficient of error (CE) of each measurement were estimated^51^. Power analysis was conducted for the results. Sample size for each experiment was chosen based on previous experience and aimed to detect at least a *p* < 0.05 in the different tests applied. No animals were excluded from the current study.

## Results

### Running exercise alleviated anhedonia induced by CUS

**Fig. 3:**
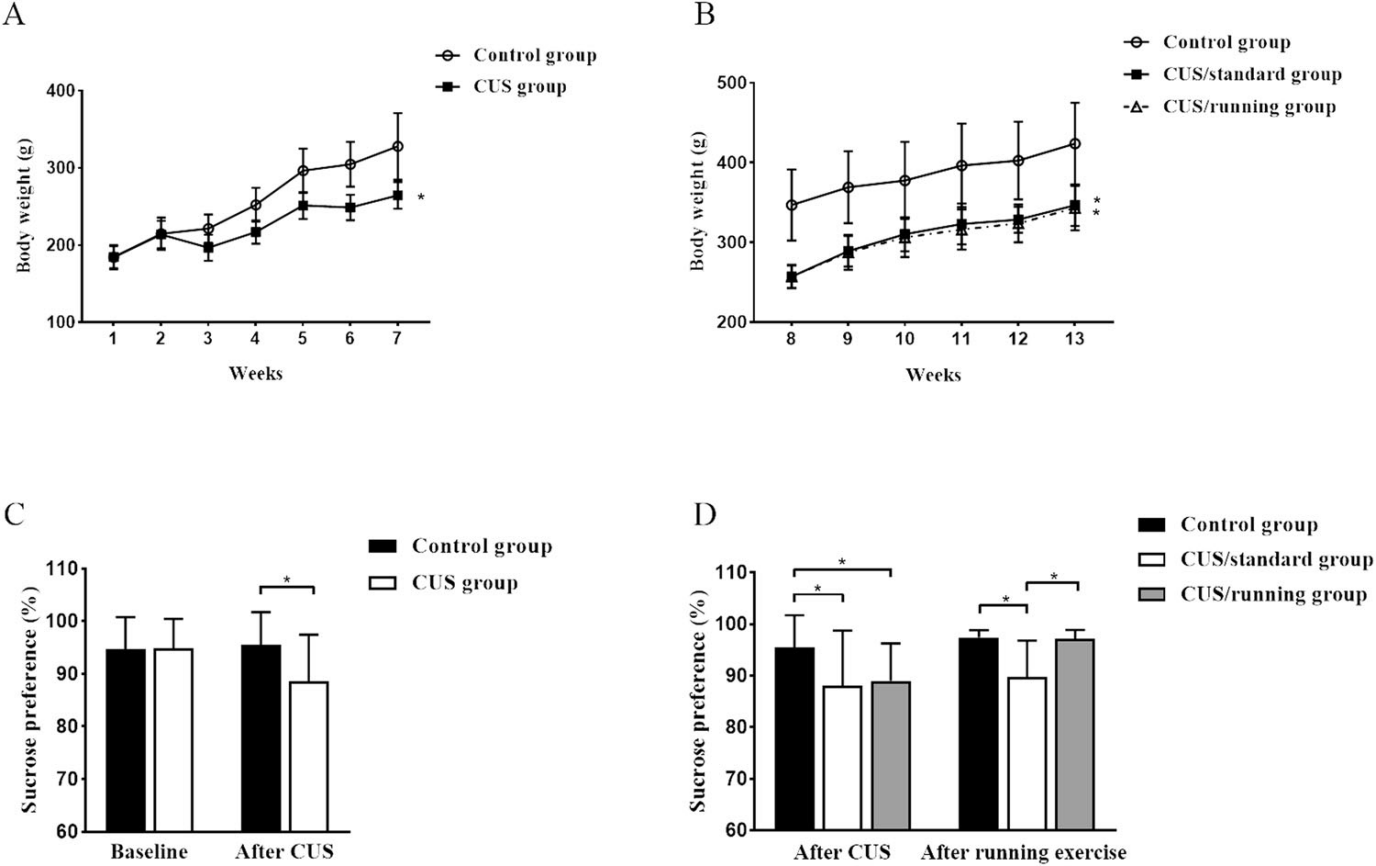
The positive effects of running exercise on the depressive-like behaviors of CUS rats. **A, B** Body weight changes in rats at different stages (mean ± SD). **A** The body weights of the control group (*n* = 23) and the CUS group (*n* = 37) during the first 7 weeks. **B** The body weights of the control group (*n* = 23), the CUS/standard group (*n* = 17), and the CUS/running group (*n* = 20) during the last 6 weeks. **C, D** The sucrose preferences of rats at different stages (mean ± SD). **C** The sucrose preferences of the control group (*n* = 23) and the CUS group (*n* = 37) at the end of CUS. **D** The sucrose preferences of the control group (*n* = 23), the CUS/standard group (*n* = 17), and the CUS/running group (*n* = 20) at the end of the running exercise period. Asterisk indicates *p* < 0.05. CUS chronic unpredictable stress.

At the beginning of this experiment, the body weight and sucrose preference of the CUS group were similar to those of the control group. From the second week of CUS intervention, the body weights of the rats in the CUS group were significantly lower than those of the rats in the control group (*p* < 0.05, Fig. 3A). During the running exercise period, the body weights of the both the rats in the CUS/standard group and those in the CUS/running group were significantly lower than the body weights of the rats in the control group, but there was no significant difference in body weight between the CUS/standard group and the CUS/running group (*p* < 0.05, Fig. 3B).

The SPT is the “gold standard” for assessing the symptoms of depression in animal models. After 5 weeks of CUS intervention, the rats in the CUS group showed a significantly lower sucrose preference than the rats in the control group (*p* < 0.05, Fig. 3C). At the end of the running exercise period, the rats in the CUS/standard group still showed a significantly lower sucrose preference than the rats in the control group, but the sucrose preference of the rats in the CUS/running group was statistically greater than that of the rats in the CUS/standard group (*p* < 0.05, Fig. 3D). There was no significant difference in sucrose preference between the control group and the CUS/running group. These results indicated that chronic stress-induced symptoms of depression and that running exercise significantly alleviated them.

### Running exercise protected against hippocampal volume loss induced by CUS

The mean hippocampal volume was (38.89 ± 3.85) mm^3^ in the control group, (34.41 ± 1.46) mm^3^ in the CUS/standard group, (40.23 ± 2) mm^3^ in the CUS/running group. These results indicated that CUS intervention significantly reduced the hippocampal volume and running exercise significantly reversed it (*p* = 0.02; *p* = 0.005, Fig. 2B). There was 0.883 power to detect an effect size of 1.02 for the hippocampal volume.

The mean volume of the CA1 region was (16.15 ± 1.6) mm^3^ in the control group, (13.54 ± 0.77) mm^3^ in the CUS/standard group, (15.8 ± 0.98) mm^3^ in the CUS/running group. The mean volume of the CA2/3 region was (10.49 ± 1.06) mm^3^ in the control group, (10.17 ± 0.62) mm^3^ in the CUS/standard group, (11.98 ± 0.76) mm^3^ in the CUS/running group. The mean volume of the DG was (12.23 ± 1.41) mm^3^ in the control group, (10.7 ± 0.63) mm^3^ in the CUS/standard group, (12.45 ± 0.59) mm^3^ in the CUS/running group. These results indicated that CUS intervention significantly reduced the volume of CA1 region and DG (*p* = 0.08; *p* = 0.042, Fig. 2C, E), and running exercise significantly increased the volume of the three hippocampal subregions (*p* = 0.018; *p* = 0.01; *p* = 0.023, Fig. 2C–E). There was no significant difference in the volume of the CA2/3 region between the control group and the CUS/standard group (*p* = 0.605, Fig. 2D). There were 0.892, 0.846, and 0.78 power to detect an effect size of 1.035, 0.967, and 0.891 for the volume of the CA1, CA2/3, and DG, respectively.

### Running exercise increased the number of astrocytes in the hippocampal subregions of CUS rats

**Fig. 4:**
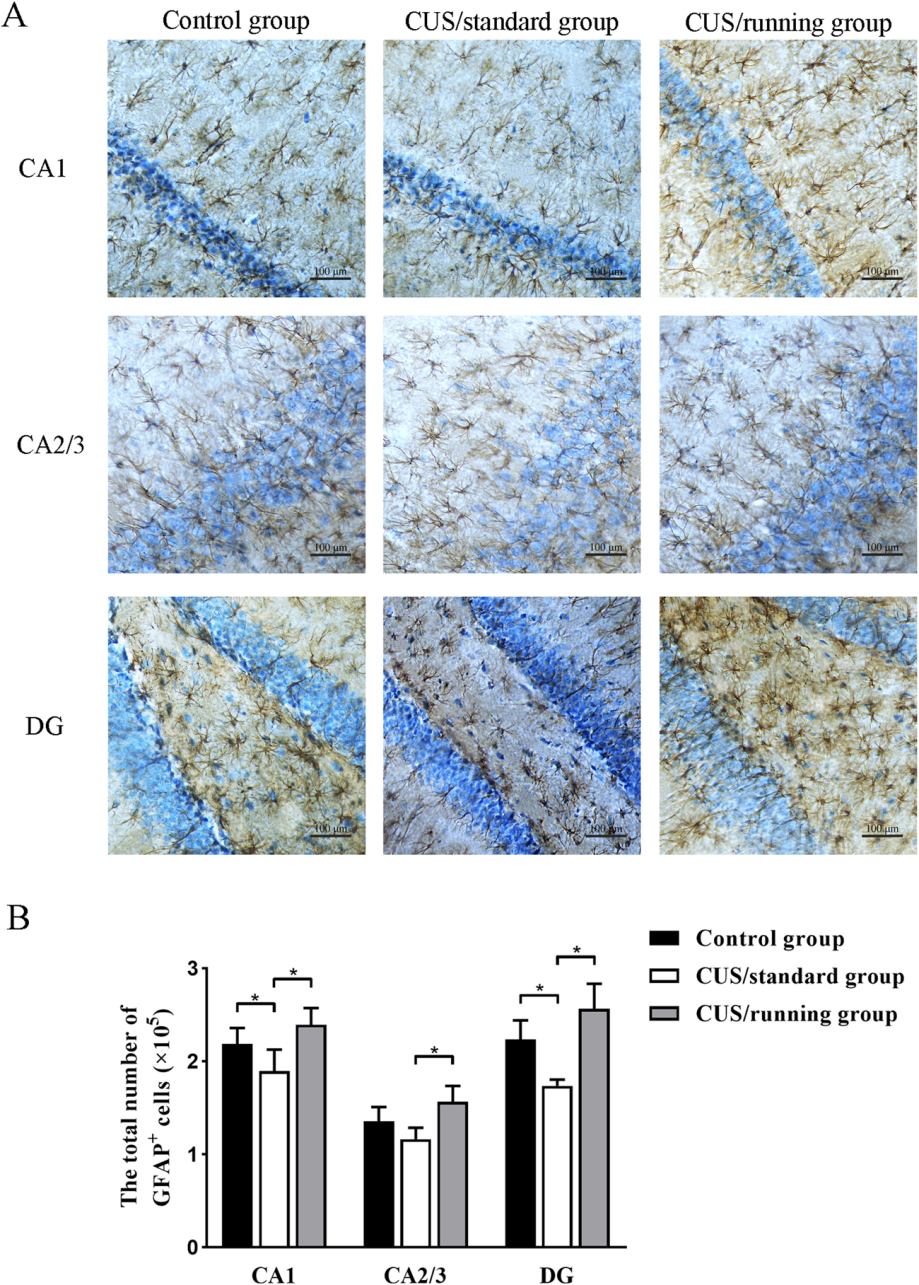
The positive effects of running exercise on the total number of GFAP^+^ cells in the hippocampus of CUS rats. **A** Representative pictures of immunohistochemical staining with an anti-GFAP antibody in the three subregions of the hippocampus in the control group, CUS/standard group, and the CUS/running group. Bar = 100 μm. **B** The total number of GFAP^+^ cells in the CA1 region, CA2/3 region and DG in the control group (*n* = 5), the CUS/standard group (*n* = 5), and the CUS/cunning group (*n* = 5) (mean ± SD). Asterisk indicates *p* < 0.05. CUS chronic unpredictable stress, GFAP glial fibrillary acidic protein.

Representative pictures of immunohistochemistry staining with an anti-GFAP antibody are shown in Fig. 4A. The mean total number of GFAP^+^ cells in the three hippocampal subregions in the three groups is shown in Table 4. These results indicated that the number of GFAP^+^ cells in the CA1 region and DG was significantly lower in the CUS/standard group than the control group (*p* = 0.036; *p* = 0.013), and significantly higher in the CUS/running group than the CUS/standard group (*p* = 0.002; *p* = 0.005). However, the number of GFAP^+^ cells in the CA2/3 area was not significantly different between the CUS/standard group and the control group (*p* = 0.067), but was significantly higher in the CUS/running group than the CUS/standard group (*p* = 0.001) (Fig. 4B). These results indicated that running exercise significantly reversed the decrease in the number of astrocytes induced by chronic stress. There were 0.959, 0.967, and 0.999 power to detect an effect size of 1.187, 1.219, and 2.101 for the number of the GFAP^+^ astrocytes in the CA1, CA2/3, and DG, respectively.

**Table 4:** The number of GFAP^+^ cell numbers.

	Control group	CUS/standard group	CUS/running group
Number of GFAP^+^ cells (×10^5^)			
CA1	2.19 ± 0.17	1.89 ± 0.23	2.4 ± 0.18
CV	0.071	0.109	0.067
CE	0.002	0.002	0.002
CA2/3	1.36 ± 0.15	1.16 ± 0.12	1.56 ± 0.17
CV	0.102	0.092	0.098
CE	0.003	0.003	0.003
DG	2.24 ± 0.21	1.74 ± 0.07	2.57 ± 0.27
CV	0.082	0.034	0.095
CE	0.002	0.002	0.002

Number of GFAP^+^ is represented as mean ± standard deviation (SD).

*CUS* chronic unpredictable stress, *GFAP* glial fibrillary acidic protein, *CV* coefficient of variation, *CE* coefficient of error.

### Running exercise increased the number of newborn astrocytes in the hippocampal subregions of CUS rats

**Fig. 5:**
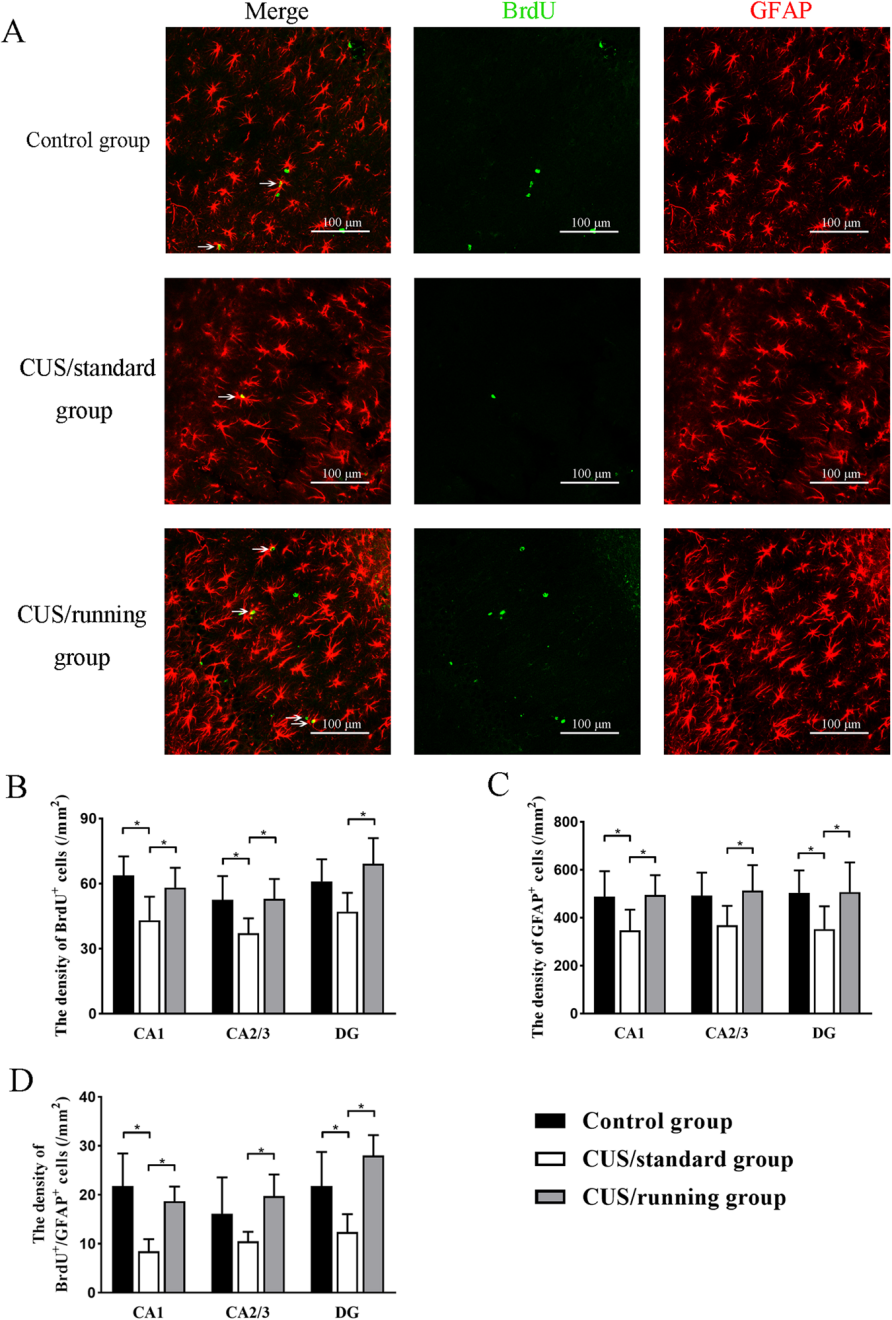
The positive effects of running exercise on the newborn astrocytes in the hippocampus of CUS rats. **A** Representative pictures of immunofluorescence staining with an anti-BrdU antibody and an anti-GFAP antibody in the hippocampus in the control group, CUS/standard group and the CUS/running group. Bar = 100 μm. **B** Quantification of BrdU^+^ cells in the three subregions of the hippocampus for the three groups of rats (*n* = 5 per group, mean ± SD). **C** Quantification of GFAP^+^ cells in the three subregions of the hippocampus for the three groups of rats (*n* = 5 per group, mean ± SD). **D** Quantification of BrdU^+^/GFAP^+^ cells in the three subregions of the hippocampus for the three groups of rats (*n* = 5 per group, mean ± SD). Asterisk indicates *p* < 0.05. CUS chronic unpredictable stress, GFAP glial fibrillary acidic protein, BrdU bromodeoxyuridine.

The variation in the number of astrocytes in the hippocampus of the rats might be due to the changes in the number of newborn astrocytes. To verify this hypothesis, we performed immunofluorescence with an antibody against BrdU, a marker of newborn cells, and an anti-GFAP antibody (Fig. 5A), and counted the number of BrdU^+^ cells, GFAP^+^ cells, and BrdU^+^/GFAP^+^ cells (Table 5).

**Table 5:** The density of BrdU^+^, GFAP^+^, and BrdU^+^/ GFAP^+^ cell.

	Control group	CUS/standard group	CUS/running group
Density of BrdU^+^ cells (/mm^2^)			
CA1	63.79 ± 7.88	43.06 ± 9.78	58.18 ± 8.2
CV	0.124	0.212	0.141
CA2/3	52.51 ± 9.8	37.18 ± 6.1	53.05 ± 8.13
CV	0.187	0.164	0.153
DG	61 ± 9.16	47.07 ± 7.78	69.17 ± 10.52
CV	0.15	0.165	0.152
Density of GFAP^+^ cells (/mm^2^)			
CA1	488.18 ± 94.81	347.28 ± 77.09	494.95 ± 74
CV	0.194	0.222	0.15
CA2/3	492.24 ± 86.13	368.18 ± 72.6	513.39 ± 95.3
CV	0.175	0.197	0.186
DG	504.28 ± 83.78	352.34 ± 85.15	506.7 ± 111.3
CV	0.166	0.242	0.22
Density of BrdU^+^/ GFAP^+^ cells (/mm^2^)			
CA1	21.82 ± 5.94	8.5 ± 2.2	18.7 ± 2.67
CV	0.272	0.259	0.143
CA2/3	16.12 ± 6.64	10.52 ± 1.72	19.72 ± 3.93
CV	0.412	0.163	0.199
DG	21.8 ± 6.21	12.43 ± 3.25	28.04 ± 3.7
CV	0.285	0.261	0.132

Density of BrdU^+^, GFAP^+^, and BrdU^+^/GFAP^+^ cells are represented as mean ± standard deviation (SD).

*CUS* chronic unpredictable stress, *GFAP* glial fibrillary acidic protein, *BrdU* bromodeoxyuridine, *CV* coefficient of variation.

After quantifying the number of BrdU^+^ cells in the three subregions of the hippocampus, we found that in the CA1 and CA2/3 areas, the density of the BrdU^+^ cells in the CUS/standard group was significantly lower than that in the control group (*p* = 0.005; *p* = 0.021). A similar trend was also found in the DG. (*p* = 0.054). Moreover, the density of BrdU^+^ cells in the three areas in the CUS/running group was significantly higher than that in the CUS/standard group (*p* = 0.030; *p* = 0.018; *p* = 0.005) (Fig. 5B). There were 0.88, 0.805, and 0.868 power to detect an effect size of 1.016, 0.918, and 0.997 for the density of the BrdU^+^ cells in the CA1, CA2/3, and DG, respectively.

The density of GFAP^+^ cells in the hippocampal subregions in the three groups (*p* = 0.033, *p* = 0.062, and *p* = 0.042 for control vs. CUS/standard in the CA1 region, CA2/3 region, and DG, respectively; *p* = 0.026, *p* = 0.033, and *p* = 0.039 for CUS/standard vs. CUS/running in the CA1 region, CA2/3 region, and DG, respectively) (Fig. 5C) as well as the density of BrdU^+^/GFAP^+^ cells were also quantified. In the CA1 area and DG, the density of BrdU^+^/GFAP^+^ cells in the CUS/standard group was significantly lower than that in the control group (*p* = 0.024; *p* = 0.013), but there was no significant difference in the density of BrdU^+^/GFAP^+^ cells between these groups in the CA2/3 area (*p* = 0.109). In the three areas, the CUS/running group showed a significantly higher density of BrdU^+^/GFAP^+^ cells than the CUS/standard group (*p* = 0.001; *p* = 0.014; *p* = 0.000) (Fig. 5D). These results indicated that running exercise had a beneficial effect on the generation of newborn astrocytes. There were 0.717, 0.631, and 0.641 power to detect an effect size of 0.83, 0.756, and 0.765 for the density of the GFAP^+^ cells in the CA1, CA2/3, and DG, respectively. There were 0.999, 0.813, and 0.996 power to detect an effect size of 1.578, 0.927, and 1.462 for the density of the BrdU^+^/GFAP^+^ cells in the CA1, CA2/3, and DG, respectively.

## Discussion

The CUS model, as a classic animal model of depression, has often been used to simulate a depressive state^52^. In the present study, rats were exposed to CUS, and the SPT was used to evaluate anhedonia, the core symptom of depression. We found that 5 weeks of CUS intervention significantly reduced body weight, sucrose preference, and hippocampal volume. These results were consistent with previous studies. For example, we previously reported the body weights of rats are decreased after CUS intervention^43,44^. Furthermore, animals exposed to CUS show a reduced preference for sucrose solutions^39,43,44^ and hippocampal volume^10^. Therefore, our results indicated that our depression animal model was successfully established. Mounting evidence from postmortem and animal studies supports the contention that alterations in the number of astrocytes in the hippocampus are closely associated with depression^19,20,53^. In our present study, using an unbiased stereological technique, we found that CUS obviously decreased the number of GFAP^+^ astrocytes in the hippocampus of rats. Similarly, Czéh et al. used a stereological method to study the changes in GFAP^+^ astrocytes in the hippocampus of male tree shrews after long-term psychosocial stress and found that the number of astrocytes in the hippocampus was consistently decreased^10^. However, there were three main differences between our study and the study by Czéh et al. First, our animal model was different from the one used by Czéh et al. We simulated a depressive state in rats by applying different stressors to generate the CUS model, which is the most widely used and typical animal model of depression^52,54^, whereas the chronic psychosocial stress paradigm used in the male tree shrews produced a depressive state by establishing a stable dominant/subordinate relationship in which the subordinates showed distinct stress-induced behavioral changes similar to those observed in depressed patients^55–57^. However, not all depressed patients suffer from the influence of dominant/subordinate relationships. Therefore, the chronic psychosocial stress model would inevitably have limitations in simulating general depressive symptoms. Second, Czéh et al. measured changes in adrenal and testis weight rather than the SPT, the “gold standard” test for assessing the core symptoms of depression in animal models, to evaluate the depressive state of animals in their study. Therefore, our results provide a more comprehensive exposition of the relationship between the number of hippocampal astrocytes and depression-like behavior. Finally, Czéh et al. found astroglial loss in the hippocampus but did not report the astroglial changes specific to each subregion of the hippocampus. As previously noted, the hippocampal formation is one of the most sensitive and susceptible regions of the CNS, and its subregions have specific structural connectivity and functional roles^21^. In this study, the number of astrocytes in each subregion of the hippocampus was accurately quantified. Our results showed that CUS reduced the number of astrocytes in the CA1 region and DG of the hippocampus, but not the CA2/3 region. Why did the changes in astrocytes differ across hippocampal subregions in depressed rats? In a clinical study in which MRI brain scans were used to detect the volume of hippocampal subregions in depressed patients and healthy control subjects, the left CA1 region emerged as a potential marker of depression^58^. Some studies have shown that the CA1 region is closely related to the pathogenesis of depression models^59,60^. Furthermore, in the CNS of adult mammals, neurogenesis mainly occurs in two regions, one of which is the DG. Adult neural stem cells can self-renew or differentiate into astrocytes or other cells in response to specific stimuli^61^. Alves et al. found that chronic stress can decrease adult neurogenesis in the DG in rats^62^, which might lead to a reduction in the number of neural stem cells that differentiate into astrocytes. Thus, we speculated that regional sensitivity to stress and the effect of stress on astrocyte formation might explain the decrease in the number of astrocytes in the CA1 region and DG in rats after CUS intervention; however, the specific causes of this decrease need to be further studied. We are the first group to study the changes in the number of astrocytes in the hippocampal subregions in CUS rats using accurate stereological techniques, and we found that the change in the number of astrocytes induced by chronic stress in each subregion of the hippocampus was different.

Both clinical studies and animal studies have demonstrated that running exercise has positive effects on depression^32,63–65^. Lapmanee et al. compared the effects of running exercise with those of common antidepressant drugs such as fluoxetine and found that both treatments alleviate anxiety- and depressive-like behaviors in stressed male rats^66^. Consistent with these studies, we found that 6 weeks of running exercise significantly reversed the decreased percentage of sucrose preference of CUS rats, indicating that running exercise improved anhedonia in our experiment. Meanwhile, we found that running exercise significantly reversed the declined hippocampal volume of CUS rats. Therefore, the results of our present study further confirmed that exercise might have a therapeutic effect on depression. It has been reported that antidepressant drugs act on astrocytes and exert their therapeutic effects by increasing ATP gliotransmission^67^. Cobb et al. also found no reduction in the density of astrocytes in the hippocampus of depressed patients taking an antidepressant drug^20^. Thus, the number of astrocytes is closely related to depression and antidepressant therapy, but whether astrocytes are involved in the antidepressant effect of running exercise is still unknown. In our study, we found that 6 weeks of running exercise also significantly increased the number of astrocytes in the three areas of the hippocampus in CUS rats, which indicated that running exercise had beneficial effects on the number of astrocytes in the hippocampus of CUS rats. Therefore, we are the first to report that changes in astrocytes might participate in the mechanism underlying the antidepressant effects of running exercise.

What causes the running exercise-induced change in the number of astrocytes in the hippocampus? We speculated that changes in the number of newly generated astrocytes might be one of the reasons for the change in the number of astrocytes in the hippocampus. In the current study, we used immunofluorescence to test this hypothesis. The density of BrdU^+^ cells was evaluated to determine the number of newborn cells, and the density of BrdU^+^/GFAP^+^ cells was measured to determine the number of newborn astrocytes. Our present results showed that 5 weeks of CUS intervention decreased the number of newborn cells in the three regions of the hippocampus and that 6 weeks of running exercise obviously increased the number of newborn cells in the three hippocampal subregions in CUS rats. Praag et al. found that voluntary wheel running increases the total number of BrdU^+^ cells in the DG in normal mice^68^, and it has been demonstrated that both forced and voluntary exercise increase the number of BrdU^+^ cells in the DG in normal rats^69^. However, the alterations in the number of newborn cells in the CA1 and CA2/3 regions and in depression after exercise are still unclear. We found that running exercise increased the number of newborn cells in the DG, CA1 region, and CA2/3 region of the hippocampus. As already noted, the DG of the hippocampus is one of the regions, in which neurogenesis occurs in the CNS of adult mammals^61^, and the process of adult neurogenesis encompasses proliferation, differentiation, migration, and functional integration into the pre-existing circuitry^70^. Therefore, we speculated that the increase in the number of newborn cells in the CA1 and CA2/3 regions might have resulted from migration of cells from the DG to these regions. Thus, our present results suggest that running exercise improves neurogenesis in the three subregions of the hippocampus in depressed rats. Although many studies have affirmed the positive effects of running exercise on newborn astrocytes in many brain regions in normal animals^37,38^, it is unclear whether running exercise can promote the generation of new astrocytes in hippocampal subregions in depression. In the current study, we found that the density of BrdU^+^/GFAP^+^ cells was reduced in the CA1 region and DG in CUS rats and that 6 weeks of running exercise significantly increased the number of newborn astrocytes in the three regions of the hippocampus in CUS rats, which indicates that newly generated astrocytes in the hippocampus play a role in the antidepressant effect of running exercise. Therefore, we speculated that newborn astrocytes in the hippocampus might be a new target for antidepressant therapy. However, it is not clear whether the increase in the number of newborn astrocytes in the hippocampus of depressed rats after running exercise was due to the increased numbers of newborn cells or the increased differentiation of newborn cells into astrocytes. In this study, we found that running exercise increased the number of newborn cells in the hippocampus. Therefore, in the future, whether newborn cells differentiate into astrocytes is more than other cells in depression models after running exercise should be studied.

In the present study, we found that running exercise could increase the number of astrocytes and the density of newborn astrocytes in the hippocampus of depressive-like rats. What might be the mechanism for the antidepression of the astrocytes increase induced by running exercise? It has been reported that the endogenous ATP released by astrocytes^17^ and the astrocyte-specific glutamate transporters^71^ might be related to the depression. Therefore, we speculated that the astrocyte increase induced by running exercise might induce antidepressant-like effects through affecting the astrocyte-related endogenous ATP and the astrocyte-specific glutamate transporters. The exact mechanism for the antidepression of the exercise-induced astrocyte increase needs to be further investigated in the future. The understanding of the mechanism might have important implications for exploring the new treatment of human depression.

In conclusion, depression is characterized by high recurrence and suicide rates. The antidepressant effect of running exercise as a behavioral intervention was further confirmed in this study. In our present study, we observed a loss of astrocytes in the CA1 region and DG of the hippocampus in depressed rats, which further confirmed that astrocytes in the hippocampus are involved in the pathological process of depression. More importantly, our present results revealed that running exercise can reverse the decreases in the number of astrocytes and the density of newborn astrocytes in the three subregions of the hippocampus in depressed rats and increase sucrose consumption. Our findings provide new insights into the role of astrocytes in depression and the antidepressant effect of running exercise, which might provide structural bases for exploring antidepressant therapy.

## Supplementary information

Supplementary material

## Supplementary information

**Supplementary Information** The online version contains supplementary material available at 10.1038/s41398-021-01216-x.
