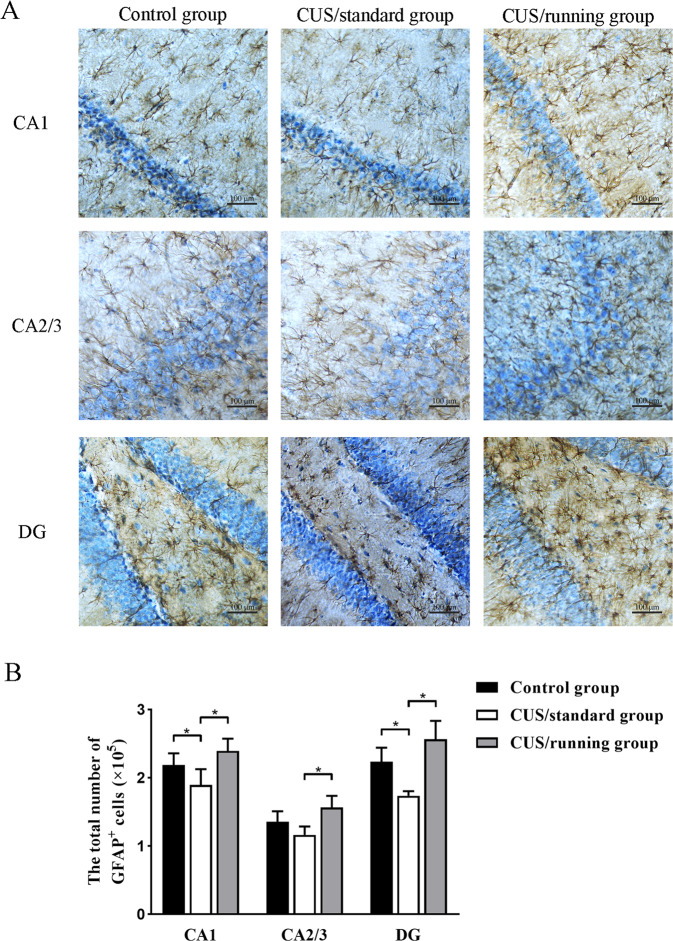# Correction: The positive effects of running exercise on hippocampal astrocytes in a rat model of depression

**DOI:** 10.1038/s41398-023-02430-5

**Published:** 2023-04-20

**Authors:** Yue Li, Yanmin Luo, Jing Tang, Xin Liang, Jin Wang, Qian Xiao, Peilin Zhu, Kai Xiao, Lin Jiang, Xiaoyun Dou, Chunxia Huang, Yuhan Xie, Yong Tang

**Affiliations:** 1 Department of Histology and Embryology, Chongqing Medical University, 400016 Chongqing, P. R. China; 2 Laboratory of Stem Cells and Tissue Engineering, Chongqing Medical University, 400016 Chongqing, P. R. China; 3 Department of Physiology, Chongqing Medical University, 400016 Chongqing, P. R. China; 4 Department of Pathophysiology, Chongqing Medical University, 400016 Chongqing, P. R. China; 5 Department of Radioactive Medicine, Chongqing Medical University, 400016 Chongqing, P. R. China; 6 Lab Teaching & Management Center, Chongqing Medical University, 400016 Chongqing, P. R. China; 7 Institute of Life Science, Chongqing Medical University, 400016 Chongqing, P. R. China

**Keywords:** Molecular neuroscience, Depression

Correction to: *Translational Psychiatry* 10.1038/s41398-021-01216-x, published online 01 February 2021

The original version of this article contained a figure error. The authors state the following: In the original file of Fig. 4A, the illustration of CA2/3 region of CUS/running group was misplaced. The authors apologize for the error.