# Supplementary material for: The positive effects of running exercise on hippocampal astrocytes in a rat model of depression
Source: Transl Psychiatry. 2021 Feb 1;11:83. doi: 10.1038/s41398-021-01216-x (PMC7851162; doi:10.1038/s41398-021-01216-x)
Supplement: Supplementary file 1 — Supplementary material [file 41398_2021_1216_MOESM1_ESM.docx]

**Figure 1**


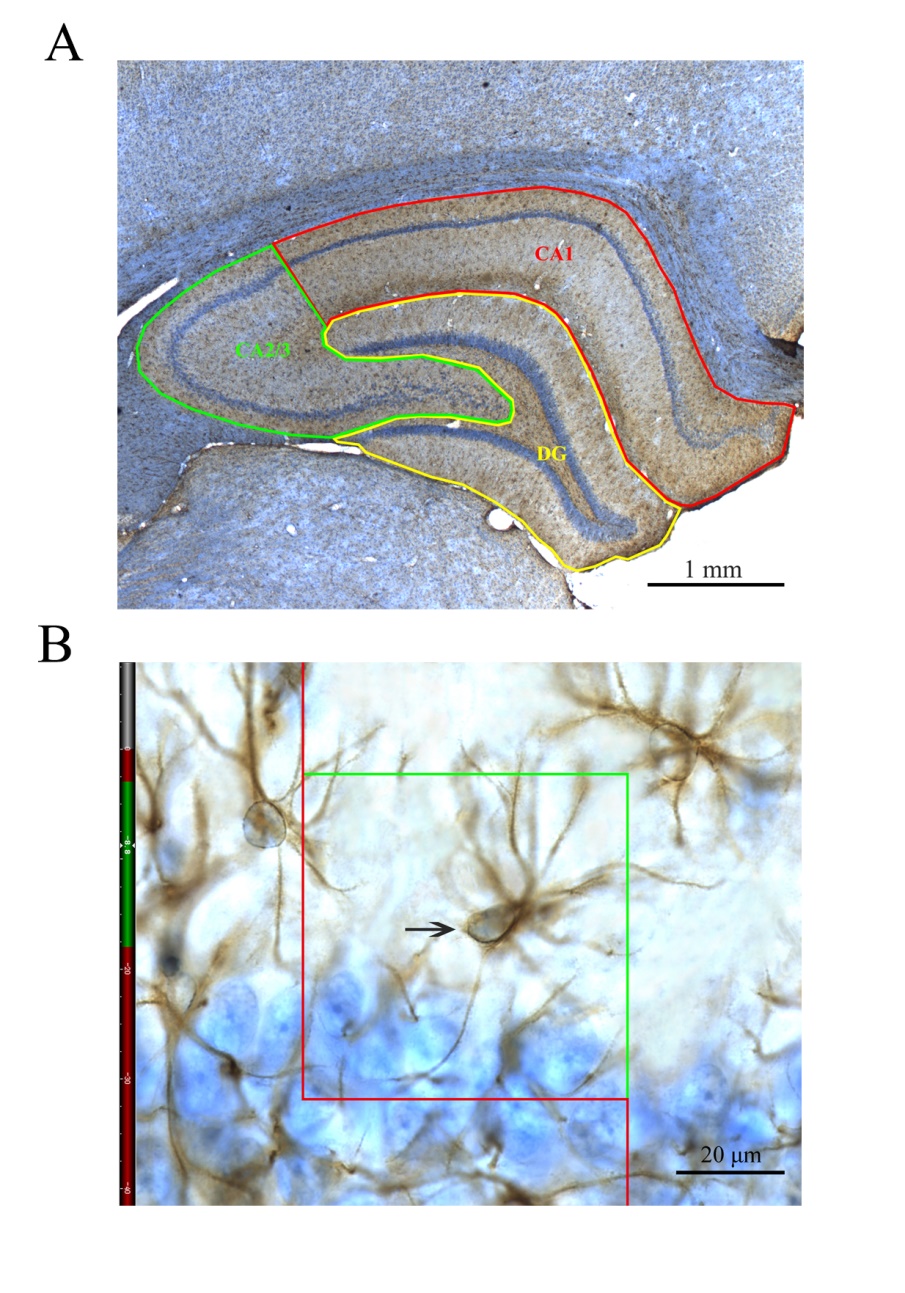


**FIGURE 1 Illustrations of the method used to quantify the number of astrocytes.**

**(A**) Schematic diagram of the subregions of the hippocampus. The CA1 region (red line), CA2/3 region (green line) and DG (yellow line) of the hippocampus were defined according to Buhl and Dann^1^. Bar = 1 mm. (**B)** A light micrograph illustrating the stereological counting frame (60 × 60 μm). The red line of the frame and its extension are the exclusion lines, and the green line of the frame is the inclusion line. GFAP^+^ cells with nuclei, but not processes, were completely or partly inside the counting frame (black arrow) but did not touch the exclusion (red) line were counted. Bar = 20 μm. GFAP: glial fibrillary acidic protein.

**References**

1 Buhl EH, Dann JF. Cytoarchitecture, neuronal composition, and entorhinal afferents of the flying fox hippocampus. *Hippocampus* 1991; **1**: 131–152.
